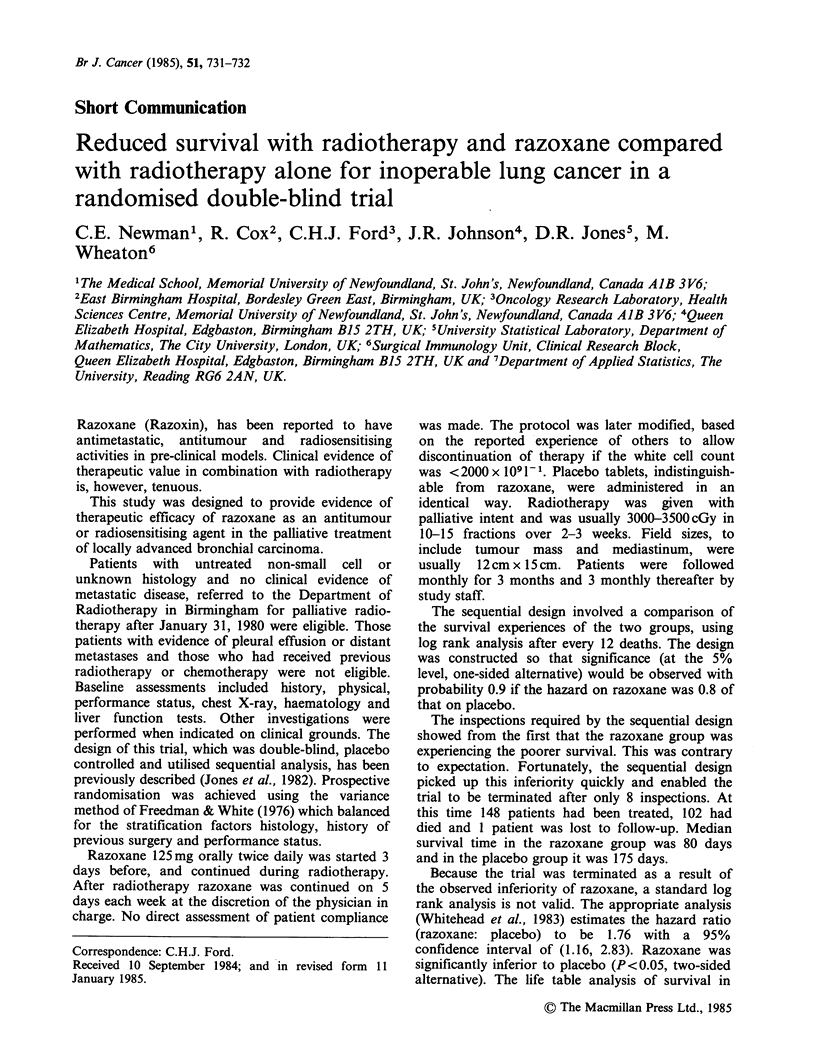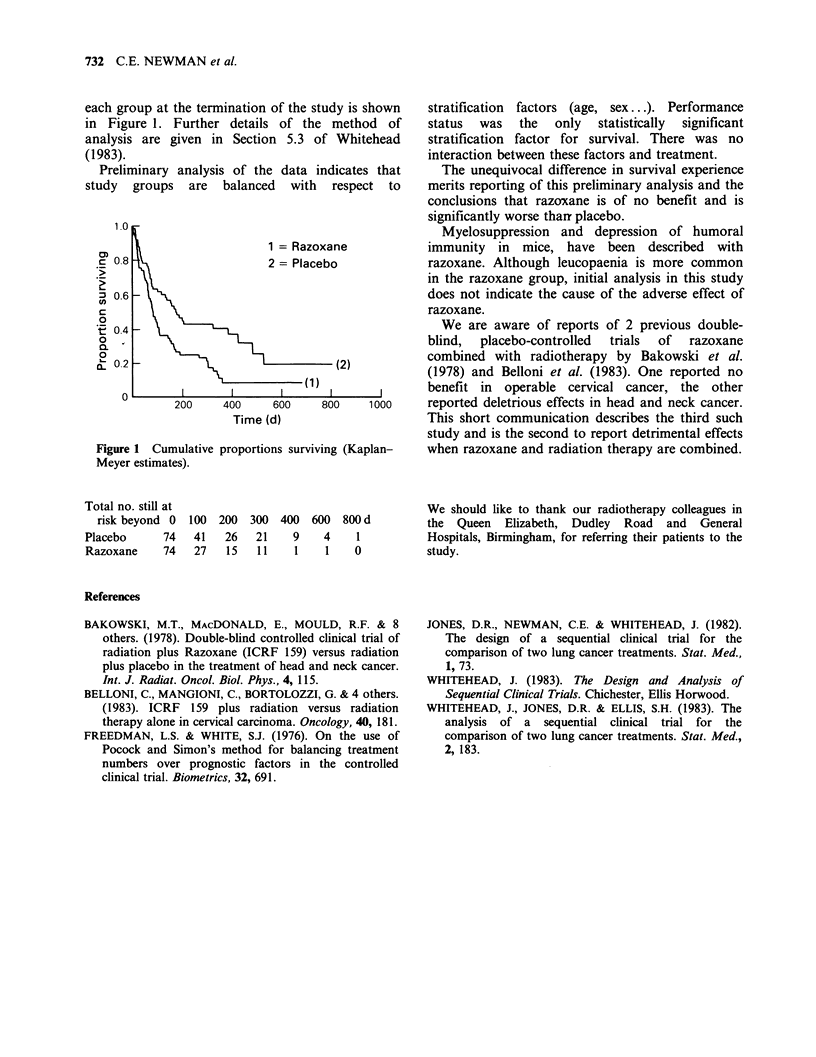# Reduced survival with radiotherapy and razoxane compared with radiotherapy alone for inoperable lung cancer in a randomised double-blind trial.

**DOI:** 10.1038/bjc.1985.109

**Published:** 1985-05

**Authors:** C. E. Newman, R. Cox, C. H. Ford, J. R. Johnson, D. R. Jones, M. Wheaton


Br J. Cancer (1985), 51, 731–732

### Short Communication

### Reduced survival with radiotherapy and razoxane compared with radiotherapy alone for inoperable lung cancer in a randomised double-blind trial

C.E. Newman[1], R. Cox[2], C.H.J. Ford[3], J.R. Johnson[4], D.R. Jones[5], M. Wheaton[6]

[1]*The Medical School, Memorial University of Newfoundland, St. John's, Newfoundland, Canada A1B 3V6;* [2]*East Birmingham Hospital, Bordesley Green East, Birmingham, UK;* [3]*Oncology Research Laboratory, Health Sciences Centre, Memorial University of Newfoundland, St. John's, Newfoundland, Canada A1B 3V6;* [4]*Queen Elizabeth Hospital, Edgbaston, Birmingham B15 2TH, UK;* [5]*University Statistical Laboratory, Department of Mathematics, The City University, London, UK;* [6]*Surgical Immunology Unit, Clinical Research Block, Queen Elizabeth Hospital, Edgbaston, Birmingham B15 2TH, UK and* [7]*Department of Applied Statistics, The University, Reading RG6 2AN, UK.*

Razoxane (Razoxin), has been reported to have antimetastatic, antitumour and radiosensitising activities in pre-clinical models. Clinical evidence of therapeutic value in combination with radiotherapy is, however, tenuous.

This study was designed to provide evidence of therapeutic efficacy of razoxane as an antitumour or radiosensitising agent in the palliative treatment of locally advanced bronchial carcinoma.

Patients with untreated non-small cell or unknown histology and no clinical evidence of metastatic disease, referred to the Department of Radiotherapy in Birmingham for palliative radiotherapy after January 31, 1980 were eligible. Those patients with evidence of pleural effusion or distant metastases and those who had received previous radiotherapy or chemotherapy were not eligible. Baseline assessments included history, physical, performance status, chest X-ray, haematology and liver function tests. Other investigations were performed when indicated on clinical grounds. The design of this trial, which was double-blind, placebo controlled and utilised sequential analysis, has been previously described (Jones *et al.*, 1982). Prospective randomisation was achieved using the variance method of Freedman & White (1976) which balanced for the stratification factors histology, history of previous surgery and performance status.

Razoxane 125 mg orally twice daily was started 3 days before, and continued during radiotherapy. After radiotherapy razoxane was continued on 5 days each week at the discretion of the physician in charge. No direct assessment of patient compliance was made. The protocol was later modified, based on the reported experience of others to allow discontinuation of therapy if the white cell count was $<2000 \times 10^9 l^{-1}$. Placebo tablets, indistinguishable from razoxane, were administered in an identical way. Radiotherapy was given with palliative intent and was usually 3000–3500 cGy in 10–15 fractions over 2–3 weeks. Field sizes, to include tumour mass and mediastinum, were usually 12 cm × 15 cm. Patients were followed monthly for 3 months and 3 monthly thereafter by study staff.

The sequential design involved a comparison of the survival experiences of the two groups, using log rank analysis after every 12 deaths. The design was constructed so that significance (at the 5% level, one-sided alternative) would be observed with probability 0.9 if the hazard on razoxane was 0.8 of that on placebo.

The inspections required by the sequential design showed from the first that the razoxane group was experiencing the poorer survival. This was contrary to expectation. Fortunately, the sequential design picked up this inferiority quickly and enabled the trial to be terminated after only 8 inspections. At this time 148 patients had been treated, 102 had died and 1 patient was lost to follow-up. Median survival time in the razoxane group was 80 days and in the placebo group it was 175 days.

Because the trial was terminated as a result of the observed inferiority of razoxane, a standard log rank analysis is not valid. The appropriate analysis (Whitehead *et al.*, 1983) estimates the hazard ratio (razoxane: placebo) to be 1.76 with a 95% confidence interval of (1.16, 2.83). Razoxane was significantly inferior to placebo ($P<0.05$, two-sided alternative). The life table analysis of survival in

---

Correspondence: C.H.J. Ford.
Received 10 September 1984; and in revised form 11 January 1985.

© The Macmillan Press Ltd., 1985

each group at the termination of the study is shown in Figure 1. Further details of the method of analysis are given in Section 5.3 of Whitehead (1983).

Preliminary analysis of the data indicates that study groups are balanced with respect to stratification factors (age, sex...). Performance status was the only statistically significant stratification factor for survival. There was no interaction between these factors and treatment.

**Figure 1** Cumulative proportions surviving (Kaplan–Meyer estimates).

| Total no. still at risk beyond | 0 | 100 | 200 | 300 | 400 | 600 | 800 d |
|---|---|---|---|---|---|---|---|
| Placebo | 74 | 41 | 26 | 21 | 9 | 4 | 1 |
| Razoxane | 74 | 27 | 15 | 11 | 1 | 1 | 0 |

The unequivocal difference in survival experience merits reporting of this preliminary analysis and the conclusions that razoxane is of no benefit and is significantly worse than placebo.

Myelosuppression and depression of humoral immunity in mice, have been described with razoxane. Although leucopaenia is more common in the razoxane group, initial analysis in this study does not indicate the cause of the adverse effect of razoxane.

We are aware of reports of 2 previous double-blind, placebo-controlled trials of razoxane combined with radiotherapy by Bakowski *et al.* (1978) and Belloni *et al.* (1983). One reported no benefit in operable cervical cancer, the other reported deletrious effects in head and neck cancer. This short communication describes the third such study and is the second to report detrimental effects when razoxane and radiation therapy are combined.

We should like to thank our radiotherapy colleagues in the Queen Elizabeth, Dudley Road and General Hospitals, Birmingham, for referring their patients to the study.

### References

BAKOWSKI, M.T., MacDONALD, E., MOULD, R.F. & 8 others. (1978). Double-blind controlled clinical trial of radiation plus Razoxane (ICRF 159) versus radiation plus placebo in the treatment of head and neck cancer. *Int. J. Radiat. Oncol. Biol. Phys.*, **4**, 115.

BELLONI, C., MANGIONI, C., BORTOLOZZI, G. & 4 others. (1983). ICRF 159 plus radiation versus radiation therapy alone in cervical carcinoma. *Oncology*, **40**, 181.

FREEDMAN, L.S. & WHITE, S.J. (1976). On the use of Pocock and Simon's method for balancing treatment numbers over prognostic factors in the controlled clinical trial. *Biometrics*, **32**, 691.

JONES, D.R., NEWMAN, C.E. & WHITEHEAD, J. (1982). The design of a sequential clinical trial for the comparison of two lung cancer treatments. *Stat. Med.*, **1**, 73.

WHITEHEAD, J. (1983). *The Design and Analysis of Sequential Clinical Trials.* Chichester, Ellis Horwood.

WHITEHEAD, J., JONES, D.R. & ELLIS, S.H. (1983). The analysis of a sequential clinical trial for the comparison of two lung cancer treatments. *Stat. Med.*, **2**, 183.